# European Patient Views on the Use of a Bio-absorbable Internal Bra in Aesthetic Breast Surgery: Insights from 2,338 Women

**DOI:** 10.1007/s00266-025-05413-7

**Published:** 2025-12-18

**Authors:** Moustapha Hamdi, Sonia Fertsch, Roy de Vita, Ernesto Maria Buccheri, Barbara Cagli, Giovanni Bistoni, Stefano Pompei, Christoph Andree

**Affiliations:** 1 Department of Plastic and Reconstructive Surgery, Brussels University Hospital – Vrije Universiteit Brussel (VUB), Brussels, Belgium; 2 Department of Plastic Surgery, Sana-Clinic, Gräulinger Str. 120, 40625 Düsseldorf- Gerresheim, Germany; 3 Department of Plastic and Reconstructive Surgery, Universität Witten/Herdecke, Witten, Germany; 4 IRCCS Regina Elena National Cancer Institute, Rome, Italy; 5 Department of Plastic Surgery UltraClinic, Via Clitunno, 22E Rome, Italy; 6 Department of Plastic, Reconstructive and Cosmetic Surgery, Campus Bio-Medico University Hospital, Rome, Italy; 7 Department of Plastic Surgery, University of Rome “Sapienza”, Rome, Italy; 8 Plastic Surgery Unit, Hospital General de Valencia, Valencia, Spain; 9 Department of Plastic Surgery, Fakeeh University Hospital, Dubai, UAE

**Keywords:** Aesthetic breast surgery, Soft tissue support, Internal bra, P4HB, GalaFLEX Scaffold

## Abstract

**Objectives:**

This study evaluated the factors considered by women who are planning to have, and who have had aesthetic breast surgery, aimed to understand the importance of cost, quantify interest and willingness to pay for Product X, a bio-absorbable internal bra (GalaFLEX™ Scaffold) and identify its most and least appreciated attributes.

**Methods:**

This was a cross-sectional, self-completed, online questionnaire in 2338 adult women from three European countries who had undergone breast surgery or were considering breast surgery. Each participant was assigned to either group 1 (previously had breast surgery) or group 2 (considering breast surgery).

**Results:**

28% of respondents had undergone at least one aesthetic breast surgery. The surgeon’s reputation and qualifications were ranked highest by majority of women in both groups. 76% of women were interested or very interested in this internal bra. 63% said they would be willing to pay an additional €1500 for the procedure if their surgeon recommended using Product X. Willingness to pay increased in-line with household income and with the amount they had paid or were considering paying for their next procedure.

**Conclusions:**

The survey showed that cost was not the primary consideration and that there was a high interest and willingness to pay for GalaFLEX^TM^. In our opinion, the use of this product could reduce the need for future revisionary surgery and therefore be more cost effective in the longer term. Based upon the study findings, we suggest incorporating the use of this internal bra in discussions with this patient population.

**Level of Evidence III:**

This journal requires that authors assign a level of evidence to each article. For a full description of these Evidence-Based Medicine ratings, please refer to the Table of Contents or the online Instructions to Authors www.springer.com/00266.

**Supplementary Information:**

The online version contains supplementary material available at 10.1007/s00266-025-05413-7.

## Introduction

Breast surgery is the most frequently performed surgical aesthetic procedure globally [1] and subsequent revision surgery is common [2–4]. A retrospective chart review (*n* = 134) by Grewal and Fisher [5] found that the most frequent reasons for revisionary surgery among aesthetic breast surgery patients were the development of ptosis (42%), capsular contracture (29%), and lower-pole deformities (19%). The loss of collagen causes skin to be thinner and less elastic, resulting in sagging and wrinkling. Other factors can accelerate this effect, such as gravity, previous surgery, weight fluctuations, pregnancy, and menopause—issues which equally affect women who have undergone aesthetic breast surgery [6, 7]. Stevens et al. [8] demonstrated that cosmetic reasons account for over 50% of reoperations of aesthetic breast surgery within the first 10 years following surgery.

Poly-4-hydroxybutyrate (P4HB) scaffold (GalaFLEX™ Scaffold; BD, Becton, Dickinson and Company, Franklin Lakes, New Jersey, USA) is used to maintain the shape and position of the breast over time following aesthetic breast surgery. GalaFLEX Scaffold is made of P4HB which has proven advantages: it is bio-absorbable, biologically derived, has been shown to demonstrate bacterial resistance and provides predictable strength and long-term support. [9–19] A low rate of adverse events and complications has been seen in many clinical studies [9, 11–19].

P4HB is notably different to other absorbable meshes as it integrates quickly and predictably into the tissue, with 75% integration at 2 weeks [20]. It also promotes the production of new collagen and is therefore replaced by the patient’s own tissue over time, leaving behind tissue which is 3 to 4 times stronger that native tissue [10, 13, 21, 22]. To the best of our knowledge, this is currently the only surgical scaffold available on the market.

The preoperative decision-making process for patients considering aesthetic surgeries is both lengthy and complex [23]. It requires significant levels of trust between the surgeon and the patient, and there is an expectation for surgeons to be clear about the financial implications of the proposed procedure [24]. However, to date and to the best of our knowledge, there has been little research into patients’ willingness to pay an additional charge for products such as GalaFLEX^TM^ Scaffold that provide long-lasting support following breast surgery and potentially prevent the need for revision surgery in the future. [25]

The objective of the study was to understand the factors that are considered important among women who are considering, and who have had aesthetic breast surgery and in particular to identify the importance of cost. We also wanted to quantify the interest and willingness of women undergoing aesthetic breast surgery to pay for GalaFLEX^TM^ Scaffold and identify which key attributes of this product are most and least appreciated by these women.

### Study Design, Methods and Patient Population

A cross-sectional, self-completed, online questionnaire study was conducted in 2338 adult women (>18 years) from three countries who had undergone at least one aesthetic breast surgery (mastopexy with or without implant, reduction or augmentation) in the past 20 years and women considering breast surgery (mastopexy with or without implant or reduction). The results of this survey are reported in-line with the Strobe statement for the reporting of cross-sectional studies [26]. The respondents were recruited from Germany, Italy, and Belgium via an online-access panel of a trusted global provider of evidence-based market and consumer research between February and May 2024. The provider conducts online market research globally and rewards panel members for answering surveys about a range of brands and products. With each survey completed, members earn reward points that can be redeemed for gift cards and PayPal credit. Email invitations to complete this survey were sent to women on the panel database, selected at random. The questionnaire was estimated to take 5–15 minutes to complete and was designed with adaptive technologies to ensure a consistent experience across desktop and mobile devices. Participants were screened for eligibility and excluded from the study if they were <18 years of age and had not undergone aesthetic breast surgery in the past 20 years or if they were not considering aesthetic breast surgery in 2024. Participants were also excluded if they did not complete the questionnaire in full and if they did not give their consent. Participants who completed the survey received remuneration equivalent to approximately €0.50.

The survey was designed to identify participants’ demographics and the type of surgery they had or were considering. Each participant was then assigned to either group 1 (women with a history of breast surgery within the past 20 years) or group 2 (women who were considering breast surgery in 2024). Demographic data collected also included marital status, household income, number of children and employment status.

**Fig. 1:**
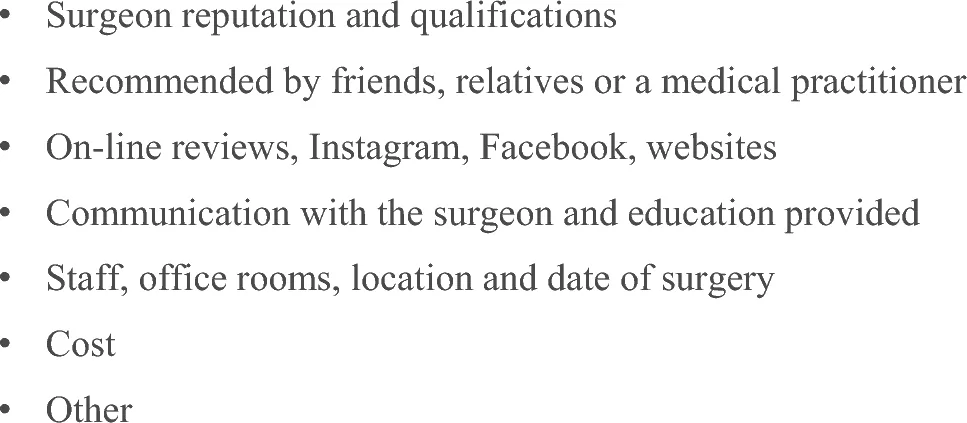
List of reasons for choosing a surgeon given to survey participants

**Fig. 2:**
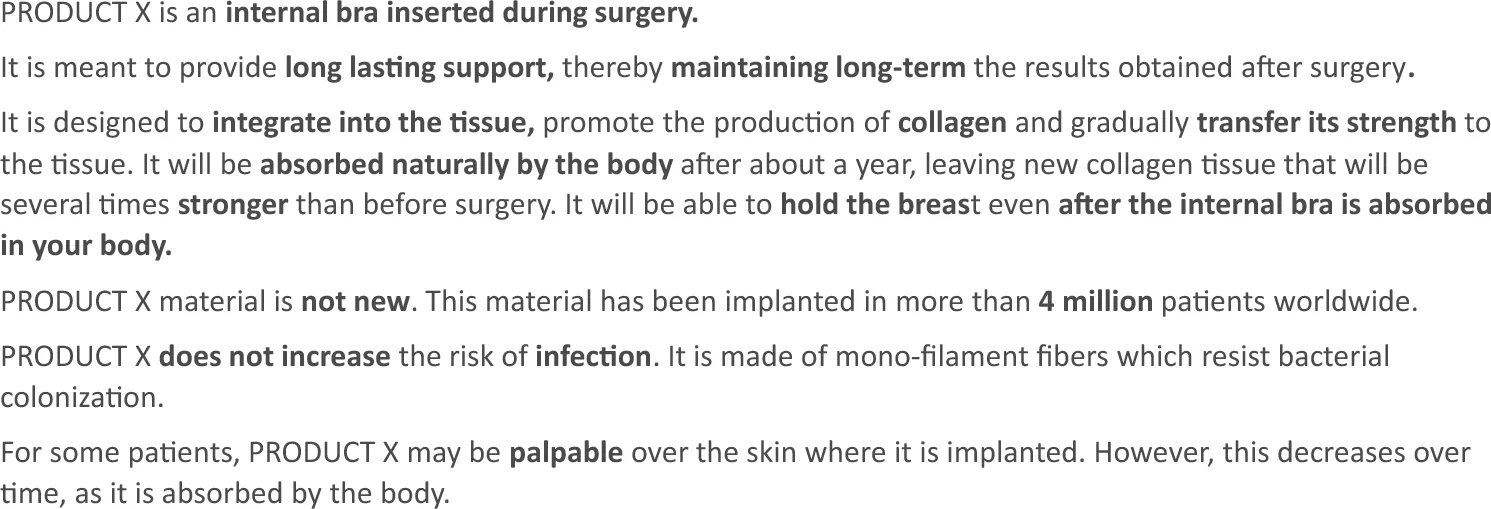
The description of Product X that survey participants were given prior to answering questions

Participants in group 1 were asked about the number and type of breast surgeries they had previously undergone, how many surgeons they saw before choosing their surgeon, the reasons for choosing that surgeon and how much they had paid for their most recent breast surgery (Fig. 1). They were then asked to rank the three most important factors they considered when choosing a surgeon for their breast surgery. They were also asked to rank their satisfaction with their most recent surgery, if they were considering future breast surgery and if so, what type of surgery that was likely to be. Participants in group 2 were asked what type of breast surgery they were considering, how advanced their plans were and how much they were willing to pay for the surgery. They were also asked what would influence their choice of surgeon and to rank the three most important factors they will consider when choosing a surgeon for their breast surgery. All participants were also given a description of GalaFLEX^TM^ Scaffold (called “Product X”) (Fig. 2) and viewed real-world before and after photographs of aesthetic breast surgery procedures utilising this product. Their interest and willingness to pay for an internal bra with the characteristics of GalaFLEX^TM^ Scaffold was then assessed.

Respondents that did not answer all the questions in the survey or who dropped out for any reason were not included in the study. The raw data were cleaned to ensure data quality, and the aggregated data were then checked for statistical significance (at 95% confidence level).

## Results

**Fig. 3:**
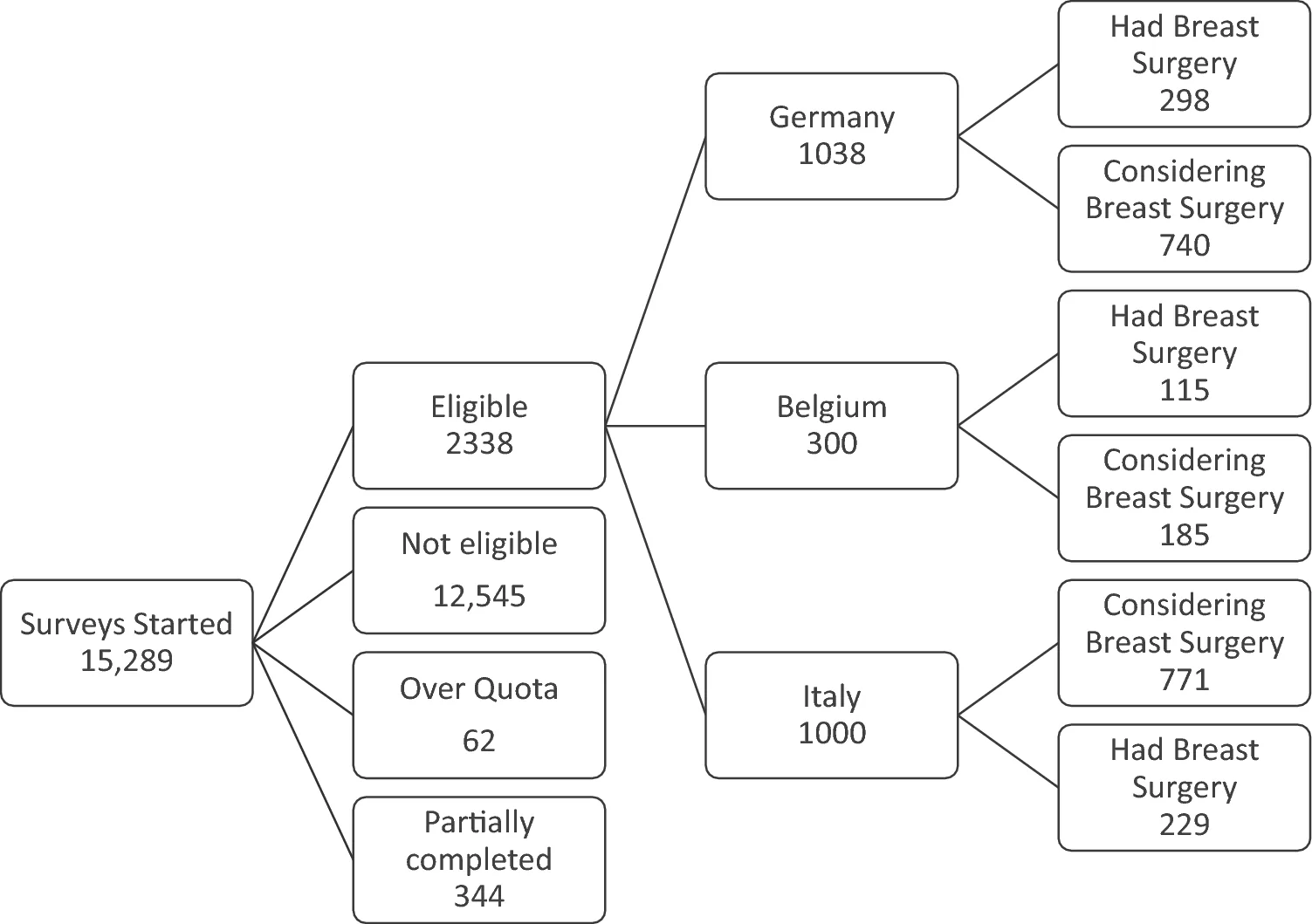
Flowchart of the selection process of the survey and the progression from surveys started to completed responses across three countries: Germany, Belgium and Italy and their status regarding whether they had undergone aesthetic breast surgery or were considering it

A total of 15,289 women from the online-access panel launched the survey. Eighty-two per cent did not meet the eligibility criteria (12,545) because they were <18 years of age, had not undergone aesthetic breast surgery in the past 20 years or were not considering aesthetic breast surgery in 2024, ~1% (175) did not consent, 2.2% (344) only partially completed the survey, and 15.3% (2338) consented and completed the questionnaire in full (Table S1). Of those, 1038 were from Germany, 1000 were from Italy and 300 were from Belgium (Fig. 3).

Demographic and participant characteristics are summarised in Table 1. Twenty-eight per cent (*n *= 642) of respondents had undergone at least one aesthetic breast surgery in the past 20 years (group 1), mainly augmentation (50%) and lift with or without implant (39%). None of these respondents’ breast surgeries was related to cancer (Table 2). The majority of women in group 1 (75%) are satisfied with their most recent surgery (76% in Italy, 67% in Belgium and 78% in Germany). However, 54% (*n *= 349) of respondents from group 1 said they had experienced some level of sagging, drooping or change in the shape of their breasts following surgery. 17% were very open to another breast surgery in the future. The most common procedures being considered by participants in group 1 were implant exchange (52%) and breast lift (47%).

**Table 1:** Demographics and participant characteristics (all patients)

Characteristic	Respondents Germany (*N* = 1038)	Respondents Italy (*N* = 1000)	Respondents Belgium (*N* = 300)	Total (*N* = 2338)
*Age (y)*				
Mean ± SD	34.87 ± 10.37 (*p*<0.05)	36.86 ± 10.6 (*p*<0.05)	37.8 ± 12.69	36.1 ± 10.85
Median (range)	34 (18–70)	36 (18–69)	37 (18–70)	35 (18–70)
Female gender, n (%)	1038 (100)	1000 (100)	300 (100)	2338 (100)
*Household monthly net income (after tax)*				
Mean (in '00 €) ± SD	29.47 ± 11.63 (*p*<0.05)	25.81 ± 11.22	27.76 ± 11.03 (*p*<0.05)	27.73 ± 11.51
*Employed, n (%)*				
Full time	558 (54)	501 (50)	133 (44)	1192 (51)
Part time	219 (21)	174 (17)	57 (19)	450 (19)
Not employed/other	261 (25)	325 (33)	110 (37)	696 (30)
*Geographic location, n (%)*				
Rural	303(29) (p<0.05)	196 (20)	118 (39) (p<0.05)	617 (27)
Suburban	369 (36)	389 (39)	114 (38)	872 (37)
Urban	366 (35) (p<0.05)	415 (41) (p<0.05)	68 (23)	849 (36)
*Marital status, n (%)*				
Single	230 (22)	186 (19)	77 (26) (*p*<0.05)	493 (21) (*p*<0.05)
In a relationship	231 (22)	147 (15)	38 (13)	443 (19)
Living with partner	169 (17)	231 (23) (*p*<0.05)	71 (24)	471 (20)
Married	336 (32)	338 (34) (*p*<0.05)	72 (25)	756 (32)
Widowed	9 (1)	15 (2)	5 (2)	29 (2)
Divorced/separated	54 (5)	45 (6)	23 (8)	122 (5)
Did not answer	9 (1)	11 (1)	4 (2)	24 (1)
*Number of children, n (%)*				
None	411 (40)	465 (46) (p<0.05)	123 (41)	999 (43)
1 child	283 (27)	245 (24)	69 (23)	597 (26)
2 children	223 (21)	222 (22)	68 (23)	513 (22)
3 children	83 (8) (*p*<0.05)	46 (5)	30 (10) (*p*<0.05)	159 (7)
≧4 children	31 (3) (*p*<0.05)	7 (1)	9 (3) (*p*<0.05)	47 (2)
Did not answer	7 (1)	15 (2)	1 (0)	23 (0)
*Bra cup size, n (%)*				
A	109 (11)	115 (11)	30 (10)	254 (11)
B	302 (29)	387 (39) (p<0.05)	71 (24)	760 (32)
C	293 (28)	280 (28)	88 (29)	661 (28)
D	188 (18) (*p*<0.05)	137 (14)	69 (23) (*p*<0.05)	394 (17)
E	130 (13) (*p*<0.05)	61 (6)	34 (11) (*p*<0.05)	225 (10)
Did not answer	16 (1)	20 (2)	8 (3)	44 (2)
*Previous breast surgery, n (%)*				
≧1 previous breast surgeries	298 (29) (*p*<0.05)	229 (23)	115 (38) (*p*<0.05)	642 (28)
No previous breast surgery	740 (71) (*p*<0.05)	771 (77) (*p*<0.05)	185 (62)	1696 (72)

**Table 2:** Characteristics and opinions of patients who had breast surgery in the past—Group 1

Characteristic	Respondents Germany	Respondents Italy	Respondents Belgium	Total
	(*N* = 298)	(*N* = 229)	(*N* = 115)	(*N* = 642)
*Number of breast surgeries*				
Mean ± SD	1.38 (0.76)	1.28 (0.92)	1.37 (0.73)	1.34 (0.82)
Median (range)	1 (1–7)	1 (1–12)	1 (1–6)	1 (1–12)
Type of breast surgery, n (%)				
Lift	57 (19)	48 (21)	19 (19)	124 (19)
Lift with implant	77 (26)	34 (15)	12 (10)	123 (19)
Reduction	59 (20)	25 (11)	50 (44)	134 (21)
Augmentation	143 (48)	131 (57)	44 (38)	318 (50)
Implant exchange or removal	14 (5)	9 (4)	9 (8)	32 (5)
Other	2 (1)	3 (1)	1 (1)	6 (1)
Years since last breast surgery				
Mean (including 0) ± SD	4.62 (4.01)	5.18 (4.41)	7.61 (5.48)	5.35 (4.57)
Median (range)	3 (0–19)	4 (0–18)	6 (0–19)	4 (0–19)
Number of surgeons consulted				
Mean ± SD	2.4 (1.52)	2.4 (2.11)	1.77 (1.3)	2.28 (1.74)
Median (range)	2 (1–12)	2 (1–25)	1 (1–11)	2 (1–25)
Reasons for choosing surgeon, n (%)				
Surgeon qualifications/reputation	190 (64)	117 (51)	60 (52)	367 (57)
Surgeon communication/education	145 (49)	87 (38)	49 (43)	281 (44)
Personal recommendation	92 (31)	97 (42)	53 (46)	242 (38)
Cost	91 (30)	36 (16)	20 (17)	147 (23)
Staff, office, location, date of surgery	92 (31)	39 (17)	16 (14)	141 (22)
Online/social media recommendation	57 (19)	33 (14)	7 (6)	97 (15)
Other	4 (1)	4 (2)	2 (2)	10 (2)
*Satisfaction immediately after most recent breast surgery (scale 0–10 where 0 = not satisfied), n (%)*				
Score 10, 9 or 8	244 (82)	170 (74)	90 (78)	504 (79)
Satisfaction today with most recent breast surgery today (scale 0-10 where 0 = not satisfied)				
Score 10, 9 or 8	233 (78)	174 (76)	77 (67)	484 (75)
*Drooping/sagging or change in the shape of breast seen since recent surgery, n (%)*				
None	149 (50)	96 (42)	48 (42)	293 (46)
Somewhat	90 (30)	88 (38)	46 (40)	224 (35)
Moderate	46 (15)	37 (16)	15 (13)	98 (15)
Very much	13 (4)	8 (4)	6 (5)	27 (4)
*Considering another aesthetic breast surgery in the future, n (%)*				
Definitely	21 (7)	9 (4)	5 (4)	35 (6)
Most likely	36 (12)	26 (11)	11 (10)	73 (11)
Possibly	100 (34)	47 (21)	29 (25)	176 (27)
Probably not	71 (24)	54 (24)	30 (26)	155 (24)
No	70 (24)	93 (41)	40 (35)	203 (32)
*Type of aesthetic breast surgery being considered in the future, n (%)*				
Implant exchange	89 (57)	39 (48)	20 (44)	148 (52)
Implant removal	8 (5)	6 (7)	1 (2)	15 (5)
Breast lift	71 (45)	43 (52)	20 (44)	134 (47)
Breast reduction	8 (5)	8 (10)	3 (7)	19 (7)
Other	6 (4)	4 (5)	1 (2)	11 (4)
Don’t know yet	12 (8)	2 (2)	4 (9)	18 (6)

Of the 73% (*n* = 1696) that had no previous aesthetic breast surgery (group 2), the majority (62%) were considering a lift with implant and over half (55%) had either chosen or were in the process of choosing a surgeon (Table 3).

**Table 3:** Characteristics and opinions of patients who had no previous breast surgery—Group 2

Characteristic	Respondents Germany	Respondents	Respondents Belgium	Total
	(*N* =740)	Italy (*N* = 771)	(*N* = 185)	(*N* = 1696)
*Type of breast surgery considered, n (%)*				
Lift	265 (36)	248 (32)	66 (36)	579 (34)
Lift with implant	475 (64)	470 (61)	103 (56)	1048 (62)
Reduction	125 (17)	166 (22)	37 (20)	328 (19)
*Current status of project, n (%)*				
Surgery scheduled	73 (10)	49 (6)	6 (3)	128 (7)
Surgeon chosen but surgery not scheduled	159 (21)	153 (20)	25 (14)	337 (20)
Consulting surgeons	168 (23)	268 (35)	33 (18)	469 (28)
Not yet consulted surgeons	340 (46)	310 (39)	121 (65)	762 (45)
*Number of surgeons consulted*				
Mean ± SD	2.4 (1.52)	2.4 (2.11)	1.77 (1.3)	2.28 (1.74)
Median (range)	2 (1-12)	2 (1-25)	1 (1-11)	2 (1-25)
*Amount willing to pay for surgery (in ‘00 €)*				
Mean ± SD	35.69 (44.86)	31.45 (43.65)	16.01 (22.91)*	31.62 (44.86)
Median (range)	20.00 (0->100)	13.00 (0-100)	0 (0-100)*	20.00 (0->100)
*Reasons for choosing surgeon, n (%)*				
Surgeon qualifications/reputation	402 (54)	524 (68)	126 (68)	1052 (62)
Surgeon communication/education	255 (35)	295 (38)	81 (44)	631 (37)
Cost	191 (26)	258 (34)	93 (50)	542 (32)
Personal recommendation	226 (36)	207 (27)	57 (31)	530 (31)
Online/social media recommendation	158 (21)	109 (14)	18 (10)	258 (17)
Staff, office, location, date of surgery	121 (16)	115 (15)	24 (13)	260 (15)
Other	30 (4)	3 (0)	4 (2)	37 (2)

*Belgian participants’ surgery potentially reimbursed by social healthcare system

Participants were asked to select all the factors that they considered important when choosing a surgeon. They were then asked to choose the 3 factors they considered the most important and rank them in order of importance.

The 3 factors that participants considered most important when choosing a surgeon were similar for both groups in all three countries. The surgeon’s reputation and qualifications were ranked in the top 3 by 57% of women in group 1 and 62% of those from group 2. Communication with the surgeon and the education they provided were rated in the top 3 by 44% of women from group 1 and 37% from group 2. Recommendations from friends, relatives or a doctor were ranked highly (in the top 3) by 38% from group 1 and 31% from group 2. Online reviews were considered least important, with just 15% of women in group 1 and 17% of women in group 2 ranking this factor as one of their top 3 considerations. Cost was considered important by more women who were considering aesthetic breast surgery (Table 3) with 32% ranking this factor in the top 3 compared to 23% of those who had previously had surgery (Table 2).

Following a review of the product attributes and viewing real-world before and after photographs of aesthetic breast surgery procedures utilising this product, the majority of patients (76%) from both groups were interested or very interested in GalaFLEX-like internal bra (Product X) (Table 4). Slightly more women who were considering breast surgery were interested/very interested (77.7% vs 72.1%). A higher proportion of women from Germany and Italy (78% and 77%, respectively, *p *= <0.05) were interested or very interested than women from Belgium (68%). Sixty-four per cent of respondents cited the ability of the product to provide long-lasting support and results even after being absorbed by the body as its most liked attribute. This attribute was liked by a higher proportion of women in Belgium (69% *p* = <0.05) than in Italy (58%). Other highly rated attributes were that it makes the breast tissue stronger (49% of respondents) and that it does not increase the risk of infection (49% of respondents). Forty-two per cent of women cited the fact that it may be palpable after surgery as the least preferred attribute.

**Table 4:** Participants views on Product X (GalaFLEX^TM^) (all patients)

	Respondents Germany	Respondents Italy	Respondents Belgium	Total
	(*N* =1038)	(*N* = 1000)	(*N* = 300)	(*N* = 2338)
*Interest in Product X (scale 1-5 where 1 = not at all interested)*				
Mean ± SD	3.98 (0.81)	3.95 (0.79)	3.71 (0.94)	3.93 (0.82)
*Features of Product X most liked, n (%)*				
Provides long-lasting support and results	707 (68.1)	576 (58)	207 (69)	1490 (64)
Absorbed naturally after about a year	553 (53)	506 (51)	121 (40)	1180 (51)
Makes new breast tissue stronger	566 (55)	431 (43)	152 (51)	1149 (49)
The material is not new	221 (21)	137 (14)	54 (18)	412 (18)
Does not increase the infection risk	526 (51)	484 (48)	135 (45)	1145 (49)
Nothing	43 (4)	23 (2)	32 (11)	98 (4)
*Features of Product X least liked, n (%)*				
No need seen	123 (12)	118 (12)	52 (17)	293 (13)
Use of foreign material	384 (34)	353 (35)	131 (44)	832 (36)
May be palpable after surgery	470 (45)	421 (42)	88 (29)	979 (42)
Other	17 (2)	10 (1)	3 (1)	30 (1)
Nothing	267 (26)	244 (24)	83 (28)	594 (25)
*Willingness to pay additional charge for GalaFLEX*^*TM*^*, n (%)*				
€ 1500	687 (66)	647 (65)	142 (47)	1476 (63)
€ 2000	568 (55)	530 (53)	99 (33)	1197 (51)
€ 3000	381 (37)	341 (34)	59 (20)	781 (33)

**Fig. 4:**
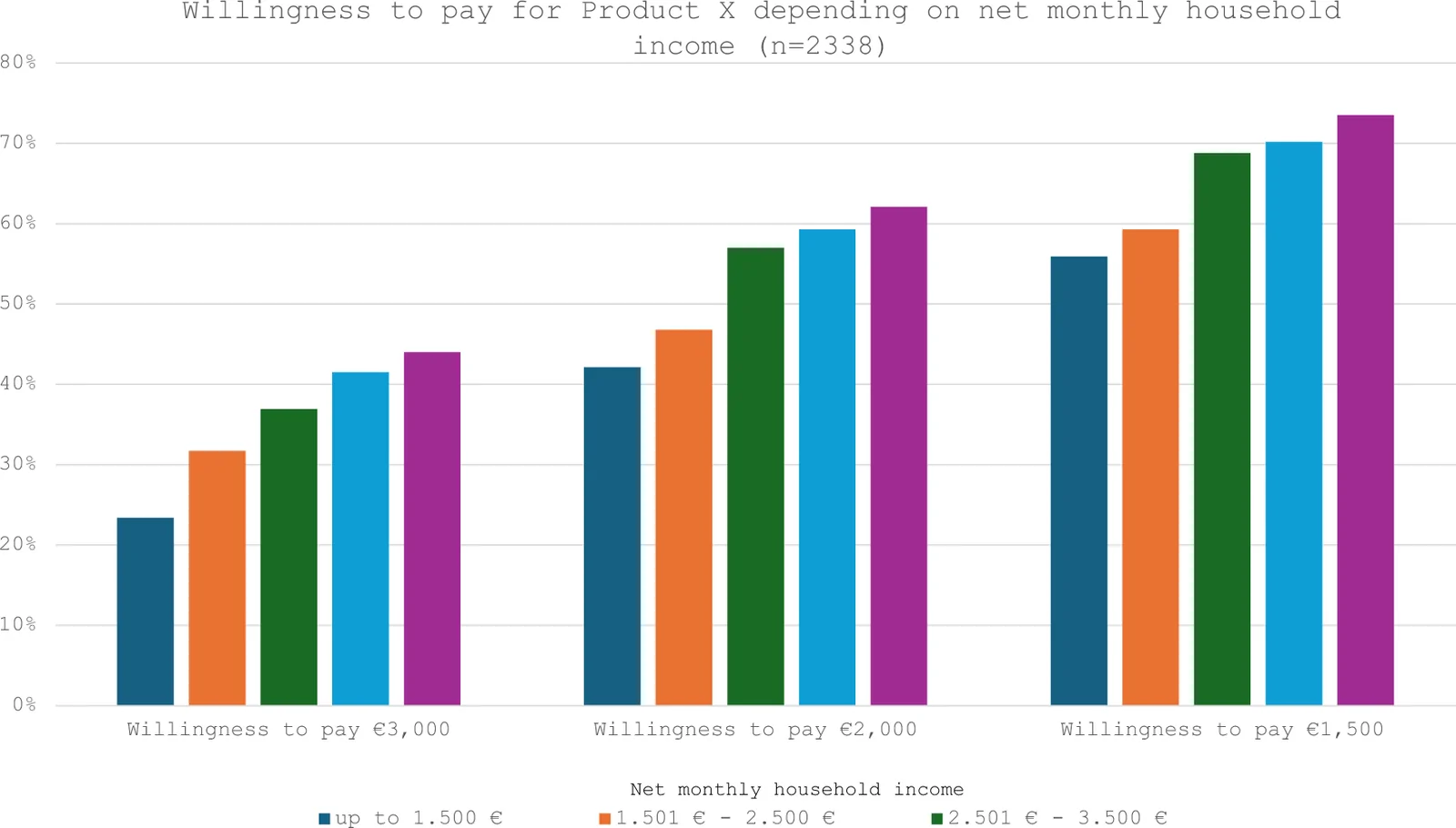
Graph showing the percentage of women (*n* = 2338) willing to pay an additional €1500, €2000 or €3000 for Product X, based on their net monthly household income

**Fig. 5:**
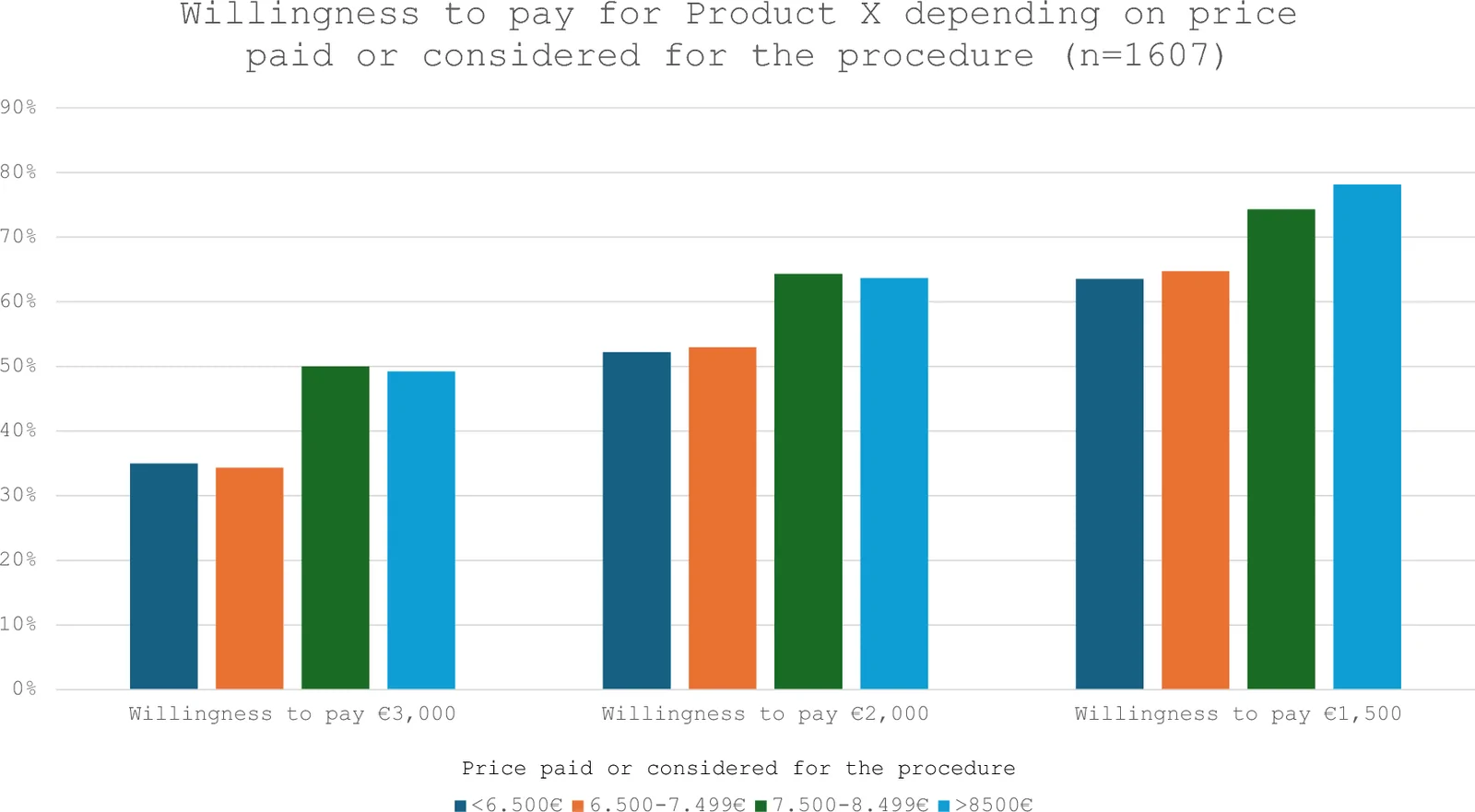
Graph showing the percentage of women (n0.=1607) willing to pay an additional €1500, €2000 or €3000 for product X depending on the amount they had paid or were planning to pay for their procedure

The majority of women responding to the survey (63%) said they would be willing to pay an additional €1500 for the procedure if their surgeon recommended using this internal bra, 51% would pay an additional €2000, and one-third (33%) said they would be willing to pay an additional €3000. Willingness to pay an additional €1500 was similar in both groups (59.2% and 64.6%); however, there were more respondents in group 1 who had breast augmentation, and more respondents in group 2 who were considering breast lift/lift with implant. Willingness to pay increased in-line with higher household income (Fig. 4**)**. They were also more likely to pay a higher price depending on how much they had paid or were considering paying for their next procedure (Fig. 5). 78% of those who had paid or were considering paying >€8,500 for the procedure were willing to pay an additional €1,500 for this internal bra to be used. Two-thirds (65%) who had paid or were considering paying <€6,500 for the procedure were willing to pay an additional €1,500 for this internal bra.

## Discussion

Our cross-sectional survey of 2338 women clearly demonstrates that cost is not the primary consideration for women when choosing a surgeon for aesthetic breast surgery. The qualifications and reputation of the surgeon were the most important factors for respondents, followed by the communication and education they had from their surgeon. The survey also showed a high interest in, and willingness to pay for an internal bra with the characteristics of GalaFLEX^TM^ Scaffold, among women undergoing aesthetic breast surgery.

Recommendations from relatives or another medical practitioner were also seen as important, whereas online reviews were only cited as important factors in the decision-making process by 15% of those who had already had breast surgery and 17% of those who were considering it.

A high proportion (≧ 49%) of women in both groups liked most of the features of this internal bra: long-lasting support and results, absorbed by the body; does not increase the risk of infection and makes the breast tissue stronger. Our survey also highlighted the importance of preoperatively discussing the possibility of palpability of the product, as 42% of women expressed dislike for this attribute.

The participants of our survey showed there was a high interest and willingness to pay regardless of income. Most women (63%) would be willing to pay an additional €1500 for GalaFLEX^TM^ internal bra to be used by their surgeon. Women who had not yet consulted a surgeon were shown to have a lower willingness to pay across the three countries. Willingness to pay either €1500, €2000 or €3000 increased in-line with higher household income in both groups, and it did not decrease, but slightly increased, with higher procedure prices.

We believe that our study confirms that the value of a surgeon’s reputation and the education they provide patients during the consultation are more important for women considering breast surgery than the one-off cost of the procedure. In our opinion, the use of this type of internal bra could be viewed as an “insurance policy”, reducing the need for revisionary surgery and being more cost effective for patients in the longer term. It is therefore reasonable to suggest that its use should be included in the preoperative discussion with all relevant patients who are considering aesthetic breast surgery.

Our findings should be interpreted bearing in mind some study limitations. Whilst clinical data demonstrating outcomes up to 45 months have been published [27], longer-term results are not yet available. The study relies on self-reported data collected via an online panel. The use of such panels could introduce selection bias as participants may not be representative of the wider population of women undergoing or considering this type of breast surgery. As the survey was relatively short, this may have impacted the depth and reliability of some of the responses, particularly those around complex issues such as willingness to pay. Whilst participants received a small monetary reward for fully completing the survey (€0.50), we do not believe that this would have had a significant impact on the answers to their questions.

Prior to viewing the real-world before and after photographs of aesthetic breast surgery procedures utilising Product X, participants were given a written explanation of the natural and external factors contributing to breast ptosis. The function, benefits and safety profile of Product X were also explained to participants. This could have created a positive framing effect.

As the results are limited to three European countries, they may not be generalisable or applicable to other settings. Cultural and healthcare system differences that may influence cost perceptions are not analysed in detail. Future studies may therefore involve a similar survey in women from other countries. In addition, a large case series to document the stability of aesthetic breast surgery with GalaFLEX^TM^ Scaffold after 5 years may also provide useful evidence to support the decision-making process for these women.

## Supplementary Information

Below is the link to the electronic supplementary material.

Supplementary file1 (DOCX 79 KB)
