# Correction: European Patient Views on the Use of a Bio-absorbable Internal Bra in Aesthetic Breast Surgery: Insights from 2,338 Women

**DOI:** 10.1007/s00266-026-06213-3

**Published:** 2026-08-20

**Authors:** Moustapha Hamdi, Sonia Fertsch, Roy de Vita, Ernesto Maria Buccheri, Barbara Cagli, Giovanni Bistoni, Stefano Pompei, Christoph Andree

**Affiliations:** 1 Department of Plastic and Reconstructive Surgery, Brussels University Hospital – Vrije Universiteit Brussel (VUB), Brussels, Belgium; 2 Department of Plastic Surgery, Sana-Clinic, Gräulinger Str. 120, 40625 Düsseldorf- Gerresheim, Germany; 3 Department of Plastic and Reconstructive Surgery, Universität Witten/Herdecke, Witten, Germany; 4 IRCCS Regina Elena National Cancer Institute, Rome, Italy; 5 Department of Plastic Surgery UltraClinic, Via Clitunno, 22E, Rome, Italy; 6 Department of Plastic, Reconstructive and Cosmetic Surgery, Campus Bio-Medico University Hospital, Rome, Italy; 7 Department of Plastic Surgery, University of Rome “Sapienza”, Rome, Italy; 8 Plastic Surgery Unit, Hospital General de Valencia, Valencia, Spain; 9 Department of Plastic Surgery, Fakeeh University Hospital, Dubai, UAE

**Correction to: Aesthetic Plastic Surgery (2025) 50:3668-3677** **https://doi.org/10.1007/s00266-025-05413-7**

The original online version of this article was revised to correct the affiliation information of coauthor Roy de Vita. The correct affiliation information is: IRCCS Regina Elena National Cancer Institute, Rome, Italy.

The original article has been corrected.

