# Supplementary material for: European Patient Views on the Use of a Bio-absorbable Internal Bra in Aesthetic Breast Surgery: Insights from 2,338 Women
Source: Aesthetic Plast Surg. 2025 Dec 18;50(10):3668–77. doi: 10.1007/s00266-025-05413-7 (PMC13219211; doi:10.1007/s00266-025-05413-7)
Supplement: Supplementary file 1 — Supplementary file1 (DOCX 79 KB) [file 266_2025_5413_MOESM1_ESM.docx]

| **Supplemental Table 1.** Participant Status | | |  | | |
| --- | --- | --- | --- | --- | --- |
| **Status** n (%) | **Respondents Germany** | **Respondents Italy** | | **Respondents Belgium** | **Total** |
| Terminated ^a^ | 4793 (80) | 6131 (84) | | 1621 (80.8) | 12545 (82.1) |
| Overquota ^b^ | 4 (0.1) | 33 (0.5) | | 25 (1.2) | 62 (0.4) |
| Qualified ^c^ | 1038 (17.3) | 1000 (13.7) | | 300 (15) | 2338 (15.3) |
| Partial ^d^ | 153 (2.6) | 132 (1.8) | | 59 (2.9) | 344 (2.2) |
| Total | 5988 (100) | 7296 (100) | | 2005 (100) | 15289 (100) |
|  |  |  | |  |  |
| Agreed to participate ^e^ | 5539 (92.5) | 7142 (97.9) | | 1903 (95) | 14584 (95.3) |
| Refused to participate | 59 (1) | 69 (0.9) | | 47 (2.3) | 175 (1.1) |
| Previously screened or dropped out | 390 (6.5) | 85 (1.2) | | 55 (2.7) | 530 (3.5) |
| Total | 5988 (100) | 7296 (100) | | 2005 (100) | 15289 (100) |

^a^ Respondents who did not meet the screening criteria (e.g. women who did not have breast surgery and who are also not considering breast surgery)

^b^ Respondents over and above a (non-targetable) quota, e.g. Women who had breast surgery – Augmentation: Not more than 20% of the sample

^c^ Respondents who qualified for and completed 100% of the survey

^d^ Respondents who dropped out of the survey at any point for a variety of reasons, e.g. lost internet connection, browser froze, lost interest

^e^ Respondents who answered “yes” to the following question. *“This survey is about cosmetic surgeries. All sensitive data collected in this survey will remain confidential in line with our privacy policy. This is a sensitive topic and something that might make some people uncomfortable. If answering questions about this topic makes you uncomfortable, feel free to close the survey now or at any point during the survey. Do you agree to participate in this study?”*
